# Risk-taking attitudes and their association with process and outcomes of cardiac care: a cohort study

**DOI:** 10.1186/1471-2261-9-36

**Published:** 2009-08-06

**Authors:** Kathryn M King, Colleen M Norris, Merril L Knudtson, William A Ghali

**Affiliations:** 1 Centre for Health and Policy Studies, Department of Community Health Sciences, University of Calgary; 2 Faculty of Nursing, University of Alberta, Alberta, Canada; 3 Division of Cardiology, University of Calgary, Calgary, Alberta, Canada; 4 Centre for Health and Policy Studies, Department of Community Health Sciences, University of Calgary, Calgary, Alberta, Canada

## Abstract

**Background:**

Prior research reveals that processes and outcomes of cardiac care differ across sociodemographic strata. One potential contributing factor to such differences is the personality traits of individuals within these strata. We examined the association between risk-taking attitudes and cardiac patients' clinical and demographic characteristics, the likelihood of undergoing invasive cardiac procedures and survival.

**Methods:**

We studied a large inception cohort of patients who underwent cardiac catheterization between July 1998 and December 2001. Detailed clinical and demographic data were collected at time of cardiac catheterization and through a mailed survey one year post-catheterization. The survey included three general risk attitude items from the Jackson Personality Inventory. Patients' (n = 6294) attitudes toward risk were categorized as risk-prone versus non-risk-prone and were assessed for associations with baseline clinical and demographic characteristics, treatment received (i.e., medical therapy, coronary artery bypass graft (CABG) surgery, percutaneous coronary intervention (PCI)), and survival (to December 2005).

**Results:**

2827 patients (45%) were categorized as risk-prone. Having risk-prone attitudes was associated with younger age (p < .001), male sex (p < .001), current smoking (p < .001) and higher household income (p < .001). Risk-prone patients were more likely to have CABG surgery in unadjusted (Odds Ratio [OR] = 1.21; 95% CI 1.08–1.36) and adjusted (OR = 1.18; 95% CI 1.02–1.36) models, but were no more likely to have PCI or any revascularization. Having risk-prone attitudes was associated with better survival in an unadjusted survival analysis (Hazard Ratio [HR] = 0.78 (95% CI 0.66–0.93), but not in a risk-adjusted analysis (HR = 0.92, 95% CI 0.77–1.10).

**Conclusion:**

These exploratory findings suggest that patient attitudes toward risk taking may **contribute to **some of the documented differences in use of invasive cardiac procedures. An awareness of these associations could help healthcare providers as they counsel patients regarding cardiac care decisions.

## Background

Prior work has revealed that there are differences in utilization of cardiac care and outcomes based on patient sex or gender [1-9], ethnicity [10-12], socioeconomic status [11,13-16], as well as geography (place of residence) [9,11,17-19]. Some have also speculated that patient preferences and approaches to decision-making are factors that contribute to the above mentioned differences [12,20-22]. Attitude toward risk-taking is a potentially interesting variable that could influence cardiac patients' preferences and approaches to decision-making, and in turn, be associated with their care and outcomes. Prosser and colleagues [23], for example, demonstrated that attitudes toward risk-taking were associated with the treatment decisions of patients with multiple sclerosis. Those who had risk-prone attitudes were less likely to adhere to a particular treatment regimen than patients who were non-risk-prone. Ayanian and Epstein [24] studied cardiac patients undergoing exercise testing to determine if potential gender differences risk-prone attitudes were associated with care decisions. They found that while there were no significant differences in the cardiac care decisions of men and women, men were more likely than women to have risk-prone attitudes. The investigators concluded that health researchers need to further explore the potential role of patients' attitudes about risk in decision-making around the use of coronary procedures.

Since the behaviour of individuals can be influenced by their beliefs about risk [25], it is possible that patients' attitudes about risk contribute to their decisions regarding health-related **decisions **and ultimately to their health outcomes. Indeed, Prospect Theory suggests that a person making a decision regarding treatment for coronary artery disease (e.g., medical therapy, percutaneous coronary intervention (PCI), or coronary artery bypass graft (CABG) surgery), for example, would weigh alternatives that involve certain risks relative to a particular goal (e.g., desire to be pain free) [26,27]. Decisions about healthcare inherently involve risk; exposing oneself to uncertain outcomes plays a key role in characterizing risk-taking [28]. Making such a treatment decision thus involves weighing elements of risk such as side effects in the case of medical therapy, or long-term recovery and potential harm in the case of CABG surgery. From the perspective of a cardiac patient, if the goal is very important, one might be willing to take significant risks to achieve that goal [27]. Yet, data from our group suggests that more than 4% of coronary angiography patients may refuse revascularization procedures (i.e., either PCI or CABG surgery) [29]. A person's attitude toward risk may thus help to explain healthcare utilization and outcomes [30]. Given that cardiac interventions are aimed at extending and improving quality of life (important goals), we hypothesized that possessing risk-prone attitudes could be associated with an individual deciding, with their physician, to undergo a coronary procedure (i.e., PCI, CABG surgery), and ultimately with survival.

## Methods

### Main Analytical Goal

**Figure 1:**
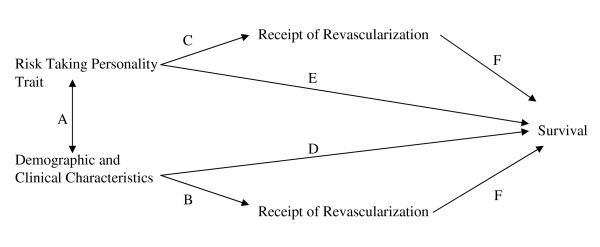
**Conceptual Model to Guide Analysis**.

We aimed to examine the associations between patients' demographic and baseline clinical characteristics, decision-making about receipt of cardiac revascularization procedures, and subsequent outcomes. The global focus of investigation was to explore the relationship between patients' risk-taking personality traits and their likelihood of undergoing cardiac procedures, and subsequent survival. To achieve this overriding goal, we needed to examine a number of inter-related associations depicted in Figure 1: (A) between patients' clinical characteristics and the risk-taking personality trait, (B) between clinical characteristics and receipt of coronary revascularization procedures, (C) between the risk-taking personality trait and receipt of coronary revascularization procedures, (D) between clinical characteristics and survival, (E) between the risk-taking personality trait and survival, and (F) also between the receipt of coronary revascularization procedures (versus medical therapy) and survival.

### Study Sample

The Alberta Provincial Project for Outcome Assessment in Coronary Heart Disease (APPROACH) is a clinical database initiative that produces detailed clinical data for all residents of Alberta, Canada undergoing cardiac catheterization in the province since 1995 [31]. The inception point for our cohort is undergoing cardiac catheterization. Thereafter, some patients receive only medical therapy, while others undergo revascularization with PCI or CABG surgery. Many (but not all) patients who are judged to be amenable to PCI undergo that procedure immediately at the same procedural sitting. This is particularly true for patients with ST elevation MI and acute coronary syndromes, but also happens in some stable angina patients. Those who undergo CABG surgery, meanwhile, would have that procedure scheduled after the results of cardiac catheterization had been reviewed by a cardiac surgeon.

One year following cardiac catheterization, patients are routinely surveyed by mailed questionnaire for collection of patient-centred data. This database represents a rich resource for studying factors related to utilization and outcomes of cardiac procedures. The APPROACH study protocol is approved annually by the Ethics Review Boards of the Universities of Calgary and Alberta and conforms to the Declaration of Helsinki.

### Main Variable of Interest – Risk Attitude

Patients registered in the APPROACH database between July 1, 1998 and December 31, 2001 were surveyed one year following their cardiac catheterization for assessment of their general (as opposed to domain specific) attitudes toward risk taking. There is considerable debate in the literature regarding measurement of risk-attitude, and specifically whether it is a general or domain-specific attribute. However, given that there is no 'gold standard' for measuring health risk attitude we, like Ayanian and Epstein [24], chose to measure general risk attitudes using the following items from the Jackson Personality Inventory: (1) "Taking risks does not bother me if the gains involved are high", (2) "I enjoy taking risks", and (3) "People have told me that I seem to enjoy taking chances" [32]. Patients were asked to respond to each item using a five point Likert scale (disagree strongly, disagree somewhat, not sure, agree somewhat, agree strongly).

### Other Variables

Demographic and clinical variables recorded in the APPROACH database at the time of cardiac catheterization include age, sex, congestive heart failure (CHF), peripheral vascular disease (PVD), chronic pulmonary disease, cerebrovascular disease, elevated creatinine (≥ 200 mmol/L), renal dialysis, diabetes, hypertension, hyperlipidemia, liver/gastrointestinal disease, malignancy/metastatic disease, prior myocardial infarction (MI), prior PCI, prior CABG surgery, prior thrombolytic therapy for MI, and smoking status (categorized as 'never', 'former', or 'current'). The indication for catheterization is recorded in one of four categories – MI within 8 weeks of catheterization, stable angina, unstable angina, or other (e.g., arrhythmias). Extent of coronary disease is recorded through documentation of coronary vessels with greater than or equal to 70% stenosis. Left ventricular ejection fraction is graded into 6 categories: < 20%, 20–34%, 35–50%, > 50%, ventriculogram not done (usually because of renal insufficiency or severely depressed cardiac function), and missing. The occurrence of revascularization procedures received within a year after catheterization is also recorded [31]. Cardiovascular team members at treatment hospitals have access to the database and prospectively enter data regarding receipt of revascularization. A survey offered at one year following cardiac catheterization also provides an opportunity to obtain more detailed sociodemographic data including household income.

Mortality (survival) data were gathered through semi-annual merging with records from the Alberta Bureau of Vital Statistics. Follow-up of patients was assessed through to December 31, 2005 (ranging from 4 to 7.5 years following cardiac catheterization and 3 to 6.5 years following completion of the survey).

### Data Analysis

Like Ayanian and Epstein [24], we assigned an ordinal scoring structure to the responses to the risk-taking items from the Jackson Personality Inventory. Each item was scored individually, assigning scores from +2 (agree strongly) to -2 (disagree strongly) with 0 representing 'not sure'. The Cronbach alpha for these questions was 0.84 in our data indicating very good internal reliability. We then followed the analysis approach used by Ayanian and Epstein [24] and averaged the available scores of the three items and dichotomized the summary variable as risk-prone (if score > 0) and non-risk-prone (if score ≤ 0). This dichotomization was judged appropriate based on a conceptual consideration of the 3 questions that constitute our risk measure, and also on the basis of exploration of the relationship between ordinally-scaled scores of -2, -1, 0, 1, and 2 in relation to use of CABG surgery after cardiac catheterization. We verified the appropriateness of our approach to averaging risk scores across items by performing a sensitivity analysis that averaged z-scores (rather than actual question scores) across items. This latter analysis yielded results virtually identical to those of our main analysis, thus leading us to only present the simpler main analysis based on the average of actual question scores.

The dichotomized composite risk variable was analyzed in our bivariate and multivariable analyses. Student t-tests and Chi-square tests were used as appropriate to compare the characteristics (demographic, clinical) and treatment methods (medical, PCI, CABG surgery) of survey responders versus non responders. Similar tests were used to compare patients who were identified as having risk-prone versus non-risk-prone attitudes. We used multiple logistic regression to identify demographic and clinical variables independently associated with risk-prone attitudes (see Figure 1, association A) and also to compare risk-adjusted use of revascularization procedures in risk-prone versus non-risk-prone patients (see Figure 1, associations B and C). We then used Kaplan-Meier plots and a Cox proportional hazards model to compare crude and risk-adjusted survival, respectively, of persons with risk-prone versus non-risk-prone attitudes (see Figure 1, associations D, E and F). Finally, we undertook additional sensitivity analyses in which we recoded the risk scores into three categories: risk-prone (mean item score > +0.5, risk-neutral (mean item score -0.5 to + 0.5) and risk-averse (mean item score < -0.5) and repeated the unadjusted logistic regression and Cox proportional hazards models described above. SPSS™ version 12 (Chicago, Illinois) was used to analyze the data.

## Results

### Specifications of Study Sample

Among surveys sent to 11841 living patients with coronary artery disease (CAD) one year following their cardiac catheterization, 2891 did not respond, 141 were unable to complete, and 2391 did not wish to complete the survey (i.e., they were unwilling to participate or asked to be removed from the study). This left 6490 patients who returned surveys. A number of patients (n = 243) did not respond to all three of the risk items. We excluded those patients who responded to only one of the three risk items. When the patient responded to two of the three items (n = 47), we calculated the mean score and included it in our analysis. Thus 6294 patients returned surveys sufficiently complete to include in our analysis, rendering a 55% useable response rate.

Patients who returned the surveys differed somewhat from those who did not, for the following clinical variables: CHF (10.2% for responders versus 12.1% for non-responders, p = .001), PVD (7.6% vs. 8.7%, p = 0.021), pulmonary disease (9.7% vs. 10.7%, p = 0.049), renal disease (1.4% vs. 2.6%, p < 0.001), diabetes (17.1% vs. 23.4%, p < 0.001), hypertension (54.9% vs. 57.2%, p = 0.008), hyperlipidemia (62.1% vs. 64.6%, p = 0.004), Liver/GI disease (3.9% vs. 3.2%, p = 0.044), previous malignancy (4.0% vs. 2.5%, p < 0.001), prior MI (46.0% vs. 54.2%, p < 0.001), former smoker (53.0% vs. 50.9%, p < 0.018), as well as current smoker at baseline (21.9% vs. 33.5%, p < 0.001).

**Figure 2:**
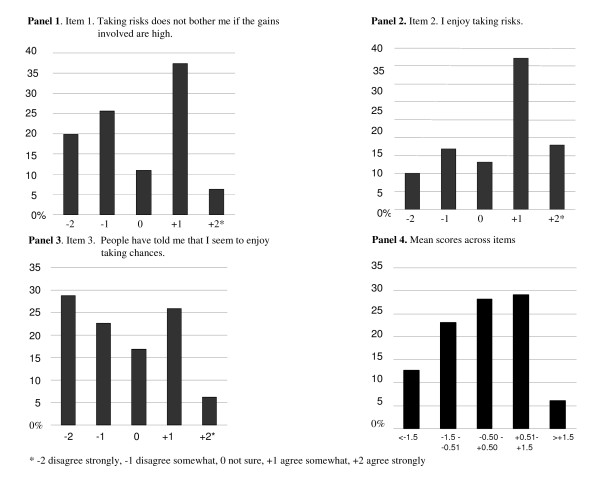
**Distribution of Ratings for Jackson Personality Inventory Risk Attitude Items and Mean Scores Across Items**.

The distribution of the Jackson Personality Inventory risk attitude item scores and the mean scores are presented in Figure 2. After dichotomizing the mean score, as described in the Methods, 45% of patients were categorized as having risk-prone attitudes.

### Patient Characteristics and their Associations with Risk Attitudes

As seen in Table 1, patients with risk-prone attitudes were on average younger and more likely to be male. The risk-prone patients were more likely to have hyperlipidemia and to be current smokers at baseline and less likely to have chronic pulmonary disease and hypertension than those with non-risk-prone attitudes. Patients with risk-prone attitudes were more likely to have 3-vessel disease while patients with non-risk-prone attitudes were slightly more likely to have single vessel disease than their counterparts. Finally, patients with risk-prone attitudes were more likely to have a higher household income than those with non-risk-prone attitudes.

**Table 1:** Demographic and clinical characteristics of sample

**Variable**	**Risk-Prone Attitudes**	**N**	**Non-Risk-Prone Attitudes**	**N**	**P**
Age (years) – Mean	61.86	(2827)	64.29	(3467)	< 0.001
Sex					< 0.001
Female	20.6%	(582)	30.8%	(1068)	
Male	79.4%	(2245)	69.2%	(2399)	
Congestive Heart Failure	9.6%	(270)	10.8%	(373)	0.12
Peripheral Vascular Disease	7.3%	(206)	7.7%	(266)	0.56
Chronic Pulmonary Disease	8.8%	(249)	10.4%	(361)	0.03
Cerebrovascular Disease	5.4%	(152)	6.5%	(225)	0.06
Elevated Creatinine	1.5%	(42)	1.3%	(46)	0.59
Renal Dialysis	0.9%	(25)	0.8%	(27)	0.65
Diabetes Mellitus	16.8%	(475)	17.1%	(593)	0.75
Hypertension	52.8%	(1494)	56.5%	(1958)	0.01
Hyperlipidemia	63.7%	(1801)	61.1%	(2118)	0.03
Liver/GI*	3.7%	(105)	4.0%	(139)	0.55
Malignancy	3.9%	(109)	4.1%	(143)	0.59
Prior MI*	46.2%	(1306)	45.5%	(1579)	0.61
Prior PCI*	8.0%	(225)	8.0%	(276)	0.99
Prior CABG* Surgery	6.9%	(195)	7.6%	(264)	0.28
Prior Lytic Therapy	4.8%	(135)	4.1%	(143)	0.21
Former Smoker	53.7%	(1517)	52.3%	(1813)	0.28
Current Smoker	24.1%	(680)	20.2%	(702)	< 0.001
Clinical Indication					0.06
Stable Angina	39.4%	(1114)	37.1%	(1286)	
Myocardial Infarction	27.9%	(790)	27.1%	(939)	
Unstable Angina	25.3%	(714)	28.0%	(972)	
Other	7.4%	(209)	7.8%	(270)	
Duke Category					0.02
1-vessel disease	18.0%	(508)	20.3%	(703)	
2-vessel disease	38.3%	(1082)	38.0%	(1316)	
3-vessel disease	34.5%	(975)	32.0%	(1109)	
left main	8.7%	(247)	8.8%	(304)	
missing	0.5%	(15)	1.0%	(35)	
Ejection Fraction					0.20
>50%	67.9%	(1920)	66.1%	(2293)	
35–50%	21.1%	(579)	22.0%	(762)	
20–34%	4.6%	(131)	4.4%	(153)	
<20%	0.7%	(21)	0.5%	(18)	
not done do to instability	3.3%	(94)	4.2%	(145)	
missing	2.3%	(64)	2.8%	(96)	
Household Income					< 0.001
<$20,000	13.2%	(374)	17.6%	(611)	
$20,000–34,999	21.2%	(598)	25.0%	(867)	
$35,000–49,999	15.9%	(450)	14.2%	(494)	
$50,000–69,999	14.4%	(406)	12.7%	(440)	
≥ $70,000	20.8%	(588)	11.4%	(395)	
Missing	14.5%	(411)	19.0%	(660)	

*GI = gastrointestinal, MI = myocardial infarction, PCI = percutaneous coronary intervention, CABG = coronary artery bypass graft

We examined the variables seen in Table 1 in logistic regression models, to identify independent associations of demographic and clinical characteristics with risk-prone attitudes in the study sample. Table 2 (first set of columns) reveals that younger age, current smoking at baseline, having unstable angina, having 3-vessel disease, and higher household income were significantly associated with having risk-prone attitudes. Notably, those who had risk-prone attitudes were nearly twice as likely to report having a household income of greater than or equal to $70,000 (CAN) per year than those who had non-risk prone attitudes. Finally, female sex was negatively associated with having risk-prone attitudes.

**Table 2:** Associations Between Patient Demographic and Clinical Characteristics with Outcomes of Interest

**Clinical Variable**	**Association with Risk-prone Attitude**		**Association with Any Revascularization**		**Association with Survival**	
**Main Variable of Interest**	**OR**	**95% CI**	**OR**	**95% CI**	**RR**	**95% CI**
Risk-prone (versus Non-Risk-Prone)			1.12	0.97–1.28	0.92	0.77–1.10
**Other Variables in Model**						
Age (10-year increments)	0.98	0.98–0.99	0.99	0.98–0.99	1.06	1.05–1.07
Female Sex	0.68	0.60–0.77	0.90	0.76–1.07	0.82	0.67–1.01
Congestive Heart Failure	1.02	0.84–1.23	0.81	0.64–1.04	1.44	1.15–1.80
Peripheral Vascular Disease	1.09	0.89–1.32	0.77	0.60–0.98	1.44	1.13–1.84
Chronic Pulmonary Disease	0.91	0.76–1.08	0.80	0.64–1.00	1.16	0.92–1.50
Cerebrovascular Disease	0.97	0.78–1.21	0.90	0.69–1.18	1.46	1.13–1.90
Elevated Creatinine	1.30	0.80–2.12	1.41	0.76–2.62	1.60	1.02–2.52
Renal Dialysis	1.01	0.54–1.88	0.33	0.17–0.66	2.17	1.19–3.97
Diabetes Mellitus	1.04	0.90–1.19	0.88	0.74–1.05	1.51	1.24–1.83
Hypertension	0.94	0.85–1.05	0.94	0.82–1.08	0.99	0.84–1.19
Hyperlipidemia	1.04	0.94–1.16	1.25	1.08–1.44	0.73	0.61–0.87
Liver/GI*	1.04	0.80–1.36	1.25	0.87–1.81	1.14	0.80–1.62
Malignancy	1.05	0.81–1.37	0.95	0.68–1.32	1.97	1.47–2.64
Prior MI*	1.02	0.89–1.16	0.68	0.57–0.80	1.34	1.09–1.64
Prior PCI*	1.01	0.83–1.22	1.18	0.93–1.50	0.94	0.70–1.26
Prior CABG* Surgery	0.88	0.71–1.07	0.26	0.21–0.33	1.04	0.79–1.36
Prior Lytic Therapy	1.14	0.88–1.47	1.14	0.80–1.62	1.06	0.72–1.57
Former Smoker	0.99	0.89–1.10			1.28	1.07–1.52
Current Smoker	1.15	1.01–1.31	1.07	0.93–1.23	1.48	1.19–1.84
						
Clinical Indication						
Stable Angina (reference)	1.00		1.00		1.00	
Myocardial Infarction	0.91	0.78–1.07	2.74	2.24–3.35	0.92	0.72–1.18
Unstable Angina	0.88	0.77–0.99	2.01	1.70–2.39	0.92	0.73–1.16
Other	0.99	0.80–1.22	0.96	0.72–1.28	1.33	0.99–1.77
						
Duke Category						
1-vessel disease (reference)	1.00		1.00		1.00	
2-vessel disease	1.10	0.94–1.28	96.72	68.41–136.73	0.18	0.87–1.60
3-vessel disease	1.26	1.07–1.49	161.60	112.62–231.88	1.54	1.14–2.10
left main	1.22	0.98–1.53	296.26	193.95–452.55	1.43	0.96–2.12
missing	0.61	0.32–1.17	20.49	10.09–41.60	2.07	0.92–4.67
						
Ejection Fraction						
> 50% (reference)	1.00		1.00		1.00	
35–50%	0.91	0.80–1.04	1.10	0.93–1.31	1.49	1.20–1.84
20–34%	1.01	0.77–1.32	0.57	0.42–.078	3.13	2.35–4.16
< 20%	1.26	0.64–2.43	0.35	0.16–0.77	3.83	2.10–6.99
not done do to instability	0.75	0.56–0.99	1.04	0.72–1.50	2.09	1.55–3.01
missing	0.84	0.59–1.18	1.72	1.08–2.77	1.64	0.98–2.59
						
Household Income						
< $20,000 (reference)	1.00		1.00		1.00	
$20,000–34,999	1.07	0.90–1.26	0.99	0.80–1.22	0.79	0.63–1.01
$35,000–49,999	1.32	1.09–1.59	1.03	0.81–1.31	0.82	0.62–1.09
$50,000–69,999	1.25	1.02–1.52	1.17	0.90–1.52	0.53	0.67–0.78
≥ $70,000	1.94	1.59–2.34	1.21	0.94–1.58	0.73	0.51–1.04
Missing	0.97	0.81–1.16	1.12	0.89–0.42	0.74	0.57–0.95
						
Treatment Received						
Medical Therapy (reference)					1.00	
CABG Surgery					0.53	0.41–0.68
PCI					0.64	0.51–0.80

*GI = gastrointestinal, MI = myocardial infarction, PCI = percutaneous coronary intervention, CABG = coronary artery bypass graft

### Associations Between Risk Attitudes and Receipt of Revascularization

Variables associated with receipt of any revascularization procedure over the year following index cardiac catheterization (Table 2, middle columns) included hyperlipidemia, myocardial infarction or unstable angina as the indication for cardiac catheterization, and 2- or 3-vessel or left main coronary disease. Variables associated with a decreased likelihood of having any revascularization procedure included increasing age, PVD, renal dialysis, prior MI, prior CABG surgery and low ejection fraction. In this adjusted model, having risk-taking attitudes were not associated with receipt of any revascularization procedure. However, in models for receipt of CABG surgery, PCI, or any revascularization procedure (see Table 3), patients who had risk-prone attitudes were more likely to have received CABG surgery than those who had non-risk-prone attitudes in both unadjusted and adjusted (for all variables shown in Table 1) models. The corresponding odds ratios were 1.21 (95% Confidence Interval [CI] 1.08–1.36) for the unadjusted model and 1.18 (95% CI 1.02–1.36) for the adjusted model.

**Table 3:** Treatment received within one year following index cardiac catheterization for risk-prone versus non-risk-prone patients (reference group)

**Variable**	**Risk-Prone Attitudes**	**N**	**Non-Risk-Prone Attitudes**	**N**	**Unadjusted OR for procedure for risk-prone versus non-risk-prone (95% CI)**	Adjusted* OR for procedure for risk-prone versus non-risk-prone (95% CI)
Any Revascularization	51.2%	(1241)	48.8%	(1185)	1.20 (1.09–1.33)	1.12 (0.97–1.28)

CABG^† ^surgery	57.0%	(931)	43.0%	(701)	1.21 (1.08–1.36)	1.18 (1.02–1.36)

PCI^†^	52.7%	(1282)	47.3%	(1150)	1.04 (0.93–1.15)	0.97 (0.85–1.09)

*The ORs presented here are adjusted for all variables listed in Table 1.

^† ^CABG = coronary artery bypass graft, PCI = percutaneous coronary intervention

### Associations Between Risk Attitudes and Survival

**Figure 3:**
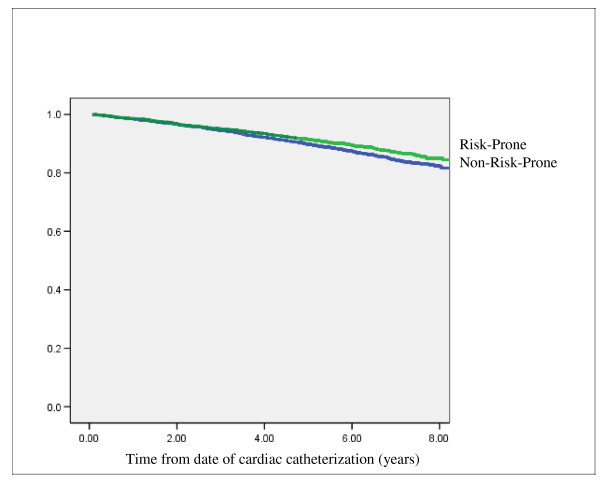
**Kaplan-Meier Plot for Survival Based on Risk Attitudes (risk-prone versus non-risk-prone)**.

Figure 3 presents a Kaplan-Meier plot of survival in risk-prone versus non-risk-prone patients. Using the date of cardiac catheterization as time zero in the survival analysis, we found that having risk-prone attitudes was associated with better survival over the follow up period, which for some patients was as long as 7.5 years (estimated survival at 7.5 years: 89% for the risk-prone group versus 85% for the non-risk-prone group; log rank test 8.02, p = 0.005). We also used Cox proportional hazards regression models to examine the unadjusted and adjusted relative risk of death in patients who had risk-prone versus non-risk-prone attitudes. The unadjusted models revealed the hazard ratio for death in patients with risk-prone attitudes was 0.78 (95% CI 0.66–0.93). When we adjusted for all the characteristics shown in Table 1, the corresponding hazard ratio was 0.92 (95% CI 0.77–1.09). When we further adjusted this model for all characteristics shown in Table 1*and *treatment received, the hazard ratio for death in patients with risk-prone attitudes was similar at 0.93 (95% CI 0.79–1.11). The apparent protective association of risk-prone attitude with survival thus became statistically insignificant after controlling for the clinical and demographic variables. However, the effect was not changed when controlling also for treatment received. Thus clinical and demographic variables, and not treatment choice, influence the association between having risk-prone attitudes and survival.

### Association Between Other Patient Characteristics, Treatment Received and Survival

Our analysis focused on risk-prone attitudes, but as seen in Table 2 (last columns) we also found that a number of clinical factors were independently associated with survival up to 7.5 years from baseline. Significant variables associated with survival were hyperlipidemia (HR = 0.73, 95% CI 0.61–0.86), and household income (HR = 0.53, 95% CI 0.67–0.78 for income $50,000–69,999). Characteristics associated with increased risk of death were: increasing age, CHF, PVD, cerebrovascular disease, elevated creatinine, renal dialysis, diabetes mellitus, malignancy, prior MI, former or current smoking at baseline, having 3-vessel disease, as well as having a low ejection fraction. Not surprisingly, receipt of revascularization procedures were associated with enhanced survival (HR = 0.53, 95% CI 0.41–0.68 for CABG surgery and HR = 0.64, 95% CI 0.51–0.80 for PCI).

### Sensitivity Analysis with a Three-Level Risk Variable

We undertook a sensitivity analysis of our findings for which we recoded the risk scores into three categories: risk-prone (mean item score > +0.5, risk-neutral (mean item score -0.5 to + 0.5) and risk-averse (mean item score < -0.5). Findings were generally similar to those of the dichotomized risk analysis. Using risk-averse patients as the reference group, patients with risk-prone attitudes and risk-neutral attitudes were more likely to have CABG surgery (OR = 1.31, 95% CI 1.15–1.50 for risk-prone; and OR = 1.15, 95% CI 0.99–1.33 for risk-neutral, respectively). Having risk-prone attitudes or risk-neutral attitudes were, meanwhile, not associated with receipt of PCI (OR = 0.99, 95% CI 0.88–1.12; and OR = 0.95, 95% CI = 0.83–1.07, respectively). There was a trend for risk-prone patients to have a reduced likelihood of mortality (HR = 0.85, 95% CI 0.70–1.04) whereas having risk-neutral-attitudes was not associated with mortality (HR = 0.98, 95% CI 0.80–1.20). Again, the findings were generally similar to those when the risk attitude scores were dichotomized, but provide additional insight in demonstrating a 'dose-response' effect – i.e., the effect was enhanced as risk attitude scores increased.

## Discussion

We have examined, using the framework presented in Figure 1, the relationships between patient characteristics (including risk taking personality trait) and the receipt of coronary revascularization, and survival. These exploratory analyses revealed that having risk-prone attitudes is associated with particular baseline patient characteristics and treatment received. Somewhat paradoxically, having risk-prone attitudes (as measured by three items from the Jackson Personality Inventory [32]) is associated with undertaking higher risk activities such as smoking at baseline and having CABG surgery (versus PCI or medical therapy). Yet, risk-prone patients did not have worse survival over 7.5 years after cardiac catheterization. In fact, they had better survival in unadjusted analyses, and a statistically insignificant hazard ratio below 1.0 in risk-adjusted survival analyses.

Inherent in considering the notion of risk is recognizing that there must be some perception of real or potential harm [25,33]. Psychologists identify that different patterns and processes of decisions are due to differences in the subjective understanding of the decision content and evaluation of the potential outcomes (i.e., risk assessment) [34-36]. Jacobs [37] for example, contends that the general public are more willing to accept 'voluntary risks' such as cigarette smoking than 'involuntary risks' such as pesticides on food, though both can be considered 'risky'. The personal importance of a decision, the familiarity with the content, and the duration of possible outcomes will influence the degree to which personal attitudes (i.e., risk-prone versus non-risk-prone) enter into the decision making process.

Possessing risk-prone attitudes has long been considered a detrimental attribute that negatively influences patients' health-related behaviors as well as their acceptance of health promoting and disease preventing recommendations [25,33,37,38]. Yet, Lantz et al.[39] suggest that individual health-risk behaviours play a stronger role in explaining health outcomes than do other variables, such as income. Based on our findings of favorable associations with risk-prone attitudes, we postulate that there may be positive aspects to having risk-prone attitudes that may, for example, enable people to succeed socially and financially, and that may lead to improved health outcomes. In the context of having CAD, possessing risk-prone attitudes may render people more prone to accepting the risk of having CABG surgery, for example.

These findings may ultimately be of relevance to healthcare providers and health system administrators. From a provider perspective, having an awareness of the patient's risk-taking attitudes in the context of health decision-making could inform how they (the providers) proceed with involving patients in healthcare decisions. As reviewed by Entwistle and Watt [40] and drawing on a perspective of 'informed choice', the patient's risk-taking preferences are highly pertinent as they undergo the cognitive and emotional information processing necessary to make health decisions. Indeed, if the provider is aware of the patient's risk-taking preferences, then the manner with which information is shared can be appropriately tailored to be of greater assistance to the patient. In this regard, the growing body of literature on optimal approaches to framing risk of both active and passive decisions in healthcare (e.g., to undergo versus forego a treatment) becomes particularly relevant [41]. Among health system administrators, who are entrusted with the challenge of offering equitable service delivery, it is similarly important to recognize that patient-centered factors such as risk-taking preferences may underlie some of the observed differences in use of invasive medical procedures. Thus, future research focusing on cardiac patients' personality traits will be worth undertaking.

Our study has limitations. The sample was self-selected, the respondents were not representative of all patients in this inception cohort, they had survived for a minimum of one year following cardiac catheterization, and may have been somewhat healthier. It is thus is possible that survey responders over-represent the proportion of patients who have risk-prone attitudes. A second limitation is that risk attitudes were assessed one year following cardiac catheterization and at an undetermined period of time following treatment choice. However, the risk-attitude personality trait may be a relatively stable construct across the adult lifespan [42-44] and its association with survival outcomes was not affected by treatment choice in our study. Further, there were some limitations to the data. We did not consider risk attitudes in a domain-specific context as would otherwise be suggested [45]. A general rather than domain-specific mechanism for categorizing risk-taking attitudes was used and the patients were not interviewed regarding their assessment of risk associated with their treatment choice. Indeed, Weber et al.[45], concluded that risk-taking behaviour is associated with individuals' perceptions of the risk versus benefits of a particular activity (e.g., having cardiac surgery, riding a bicycle without a helmet, making a particular investment) rather than in their general attitudes toward risk. Further, we did not measure health goals of respondents. Thus, we can not relate our study findings to Prospect Theory. Finally, it is not possible to know what other potential personality traits or factors may have influenced this intriguing finding. Nevertheless, there was a range of attitudes portrayed in the responses to risk attitude questions from the Jackson Personality Inventory [31], gender differences were identified, and the analyses involving the risk-taking variable specified for this study have face validity and intriguing associations with process of care and outcomes.

## Conclusion

The limitations notwithstanding, our study yields intriguing information regarding the potential associations between patients' risk-taking attitudes and aspects of their medical condition and care. Our findings reveal that there may be both positive and negative aspects to being 'risk-prone' and individual personality traits may play an important role in health outcomes in cardiac care. Further validation of these findings and an awareness of these factors could help healthcare providers in the counseling of patients regarding decisions in their cardiac care [41]. Furthermore, such factors may be found to underlie some of the observed differences in use of invasive medical procedures.

## Abbreviations

APPROACH: Alberta Provincial Project for Outcome Assessment in Coronary Heart Disease; CABG: Coronary Artery Bypass Graft; CHF: Congestive Heart Failure; CI: Confidence Interval; GI: Gastro-intestinal; HR: Hazard Ratio; MI: Myocardial Infarction; OR: Odds Ratio; PCI: Percutaneous Coronary Intervention; PVD: Peripheral Vascular Disease.

## Competing interests

The authors declare that they have no competing interests.

## Authors' contributions

All authors have contributed substantively to the development of this manuscript thus warrant inclusion as authors (conception-KMK, WAG; protocol development-KMK, CMN, MLK, WAG; data acquisition-CMN, MLK, WAG; data analysis and interpretation-CMN, WAG, KMK; manuscript development and/or critical appraisal-KMK, CMN, MLK, WAG). The authors have collectively approved the final manuscript.

## Pre-publication history

The pre-publication history for this paper can be accessed here:


